# Influence of patient's physiologic factors and immobilization choice with stereotactic body radiotherapy for upper lung tumors

**DOI:** 10.1120/jacmp.v15i5.4931

**Published:** 2014-09-08

**Authors:** Terence T. Sio, Andrew R. Jensen, Robert C. Miller, Luis E. Fong de los Santos, Christopher L. Hallemeier, Nathan R. Foster, Sean S. Park, Heather J. Bauer, Kenneth Chang, Yolanda I. Garces, Kenneth R. Olivier

**Affiliations:** ^1^ Department of Radiation Oncology Mayo Clinic Rochester MN; ^2^ Division of Biomedical Statistics and Informatics Mayo Clinic Rochester MN USA

**Keywords:** body mass index, immobilization, pulmonary function test, stereotactic body radiation therapy, upper lung tumors

## Abstract

The purpose of the present study was to compare the impact of pulmonary function, body habitus, and stereotactic body radiation therapy (SBRT) immobilization on setup and reproducibility for upper lung tumor. From 2008 through 2011, our institution's prospective SBRT database was searched for patients with upper lung tumors. Two SBRT immobilization strategies were used: full‐length BodyFIX and thermoplastic S‐frame. At simulation, free‐breathing, four‐dimensional computed tomography was performed. For each treatment, patients were set up to isocenter with in‐room lasers and skin tattoos. Shifts from initial and subsequent couch positions with cone‐beam computed tomography (CBCT) were analyzed. Accounting for setup uncertainties, institutional tolerance of CBCT‐based shifts for treatment was 2, 2, and 4 mm in left–right, anterior–posterior, and cranial–caudal directions, respectively; shifts exceeding these limits required reimaging. Each patient's pretreatment pulmonary function test was recorded. A multistep, multivariate linear regression model was performed to elucidate intervariable dependency for three‐dimensional calculated couch shift parameters. BodyFIX was applied to 76 tumors and S‐frame to 17 tumors. Of these tumors, 41 were non–small cell lung cancer and 15 were metastatic from other sites. Lesions measured <1(15%), 1.1 to 2 (50%), 2.1 to 3 (25%), and >3(11%) cm. Errors from first shifts of first fractions were significantly less with S‐frame than BodyFIX (p<0.001). No difference in local control (LC) was found between S‐frame and BodyFIX (p=0.35); two‐year LC rate was 94%. Multivariate modeling confirmed that the ratio of forced expiratory volume in the first second of expiration to forced vital capacity, body habitus, and the immobilization device significantly impacted couch shift errors. For upper lung tumors, initial setup was more consistent with S‐frame than BodyFIX, resulting in fewer CBCT scans. Patients with obese habitus and poor lung function had more SBRT setup uncertainty; however, outcome and probability for LC remained excellent.

PACS number: 89.20.‐a

PACS number: 89.20.-a

[a] Corresponding author: Kenneth R. Olivier, Department of Radiation Oncology, Mayo Clinic, 200 First St. SW, Rochester, MN 55905, USA; phone: (507) 284 8227; fax: (507) 284 0079; email: olivier.kenneth@mayo.edu

## I. INTRODUCTION

Stereotactic body radiation therapy (SBRT) is highly effective for controlling early-stage primary and oligometastatic cancers in the thorax.[1-4] SBRT for early non–small cell lung cancer (NSCLC) delivers large doses to a small area, usually in ≤ 5 fractions.[5] Conformation of high doses to the target and achieving rapid fall-off doses are critical in minimizing toxicity to healthy tissue. Effective immobilization strategy and precise target localization are important.

One challenging anatomical area for body immobilization is the apical portion of thorax and lungs.[6,7] Due to the technical requirement of delivering highly precise treatment with SBRT, respiratory motion and management are important issues to consider. Clinically, patient factors, such as body habitus and preradiotherapy cardiopulmonary reserve, are capable of influencing the degree of respiratory motion during treatment. We compared setup accuracy of two immobilization systems, S-frame and BodyFIX, to evaluate the effect of such patient-related factors as body habitus and pulmonary function on positioning errors in SBRT for upper lung tumors.

## II. MATERIALS AND METHODS

Between April 2008 and November 2011, a prospective SBRT database was searched for patients with upper lung tumor, defined as entire internal tumor volume (respiratory motion included) at or superior to the T5 vertebra. Inclusion criteria were age ≥ 18 years and diagnosis of primary or metastatic tumor in the apical lung. Patients with suspected locally confined recurrent NSCLC were not required to have biopsy confirmation because of poor pulmonary reserve and pneumothorax risk.

At our institution, patients receiving lung SBRT are treated daily, in a well-tolerated regimen with acceptable early toxicities.[8] Patients were typically offered a daily fractionation schedule. Rarely, if the patient had severe cardiopulmonary comorbidities or unusual logistical consideration, an alternative schedule could be chosen at the discretion of the treating radiation oncologist. If tumor motion ≥ 5 mm is detected at four-dimensional simulation, a method to account for motion is advised through, for example, addition of internal target volume. Typically, the planning target volume was created from internal target volume with 0.5 cm expansion in all directions to account for intrafractional uncertainties. Breath-hold or respiratory gating was not allowed and internally implanted markers were not used. Inhomogeneity corrections were used. Multiple three-dimensional beams of 6 to 10 MV were required, with either three-dimensional conformal (typically in coplanar arrangement) or intensity-modulated (only 6 MV allowed) beam planning techniques. The Mayo Clinic Institutional Review Board approved the study.

### A. Immobilization setup and data collection

Initially, full-length BodyFIX (Elekta, Stockholm, Sweden) was used for all patients receiving lung SBRT. However, pretreatment imaging showed that some patients with upper lung tumors were not sufficiently immobilized with BodyFIX. Subsequently, the extended thermoplastic S-frame was provided as an option (Fig. 1, top panel). The extended thermoplastic S-frame (also called Type-S frame) included both head and shoulder regions for reproducibility and patient comfort and was commonly used in our clinic for nonstereotactic radiotherapy treatments in patients with head and neck tumors. The BodyFIX (Fig. 1, bottom panel) was capable of reproducing accurate patient positioning between fractions and maintained a high level of intrafractional stability with vacuum application during treatment planning and actual radiotherapeutic delivery of SBRT. A thin transparent plastic sheet was used to cover the patient's body. Logistically, the position and markings of BodyFIX were also easily indexed in relation to the treatment table top associated with the linear accelerator, providing a good management system for clinical radiation therapists to use daily. For both immobilization devices, typically the patient's arms were positioned downward. No patient was immobilized with both devices

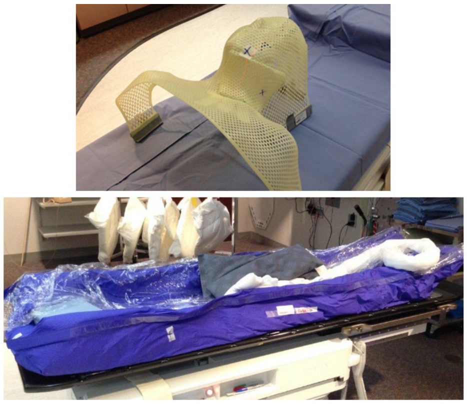

Fig. 1. Immobilization strategies for patient undergoing stereotactic body radiotherapy for upper thoracic tumors: (top) thermoplastic S-frame; (bottom) full-length BodyFIX.

simultaneously for any treatment. The number of cone-beam computed tomographic (CBCT) scans required before reaching treatment tolerance was recorded.

We performed a detailed review of patient charts and dosimetric planning documents. SBRT characteristics were recorded, including total dose, hypofractionation schedule, and treatment number. Pretreatment pulmonary function test (PFT) results, height, and weight were recorded. Age at diagnosis, chronic obstructive pulmonary disease (COPD) status, COPD severity (clinically significant COPD includes moderate, severe, and oxygen-dependent), and smoking status were accessed. Tumor status was recorded as primary, recurrent, or metastatic lung cancer from another site. The most recent PFT before the SBRT start date was recorded, if available. PFT parameters included total lung capacity, vital capacity, forced expiratory volume in the first second of expiration ($FEV_1$), residual volume, normalized $FEV_1$ to age- and sex-adjusted control, normalized ratio of $FEV_1$ to forced vital capacity (FVC), and diffusing capacity of lung for carbon monoxide.

At simulation, free-breathing four-dimensional computed tomography (CT) was performed without respiratory gating or breathing control. For initial treatment, patients were first set up to isocenter with in-room lasers and skin tattoos. Shifts from this initial couch were adjusted through CBCT. Institutional tolerance of CBCT-based shifts for treatment was 2, 2, and 4 mm in left–right, anterior–posterior, and cranial–caudal directions, respectively; shifts exceeding these limits required reimaging. The shifts were applied and a second CBCT scan was obtained for treatment position verification and reevaluation. Shift changes were measured again; if tolerance was still not met, a third CBCT scan was performed. Couch position from the first fraction treatment was used as the initial couch position for the other fractions. Couch positions were extracted from the treatment record and the magnitude of couch shifts calculated. Shift magnitude was determined by the square root of the sum of squares in X, Y, and Z directions.

Most patients — except one patient who received one 34 Gy fraction — received 3, 4, or 5 SBRT fractions. Within each fraction, the magnitude of each couch shift was quantified. Couch difference from initial position to after the first CBCT alignment was termed first shift (S1). Between first and second CBCT scans, second shift (S2) was recorded. Similarly, third shift (S3) was measured between second and third CBCT scans, if necessary. Shift errors referred to the corrections needed for couch position adjustments. No treatment fraction required more than three shifts.

## B. Statistical methods

Descriptive statistics were used to summarize patient and treatment characteristics. A conservative post hoc power analysis based on F1, S1 differences estimated that the powers needed to detect a 30% mean difference in proportions (BodyFIX = 0.6; S-frame = 0.3) were 63.1% and 74.6% at two-sided $\alpha$ values of 0.05 and 0.10, respectively. The $\chi^2$ and Wilcoxon rank sum testings were used to measure the association between categorical variables; Wilcoxon rank sum tests were used to test for association between binary variables and continuous clinical and patient characteristic variables. Spearman rank correlation coefficient was used to measure linear association of continuous variables, and scatter plots were used to visualize univariate relations. Box plots were used to graphically show differences among error variables by S-frame versus BodyFIX.

Local control (LC) was calculated as months from the beginning of SBRT to local relapse for local (primary or recurrent) and metastatic cases. Progression-free survival (PFS) was calculated as months from date of diagnosis to local or metastatic relapse or death. Overall survival was calculated as months from diagnosis date to death or last contact. The Kaplan-Meier and univariate Cox proportional hazards models were used. Multivariate regression models were used to assess the relation between error and shift variables with immobilization method after adjusting for body habitus-related (body mass index [BMI] or body surface area [BSA]) and other significant PFT variables from univariate analyses. Statistical tests were two-sided, with $p < 0.05$ considered significant. Statistical analyses were performed with SAS software (SAS Institute Inc., Cary, NC).

## III. RESULTS

### A. Patient and tumor characteristics

Eighty-eight patients met our search criteria. Median follow-up was 8.7 months (quartiles, 4.4–19.1 months). Median follow-up period for living patients was 11.2 months. Two patients received SBRT to synchronous tumors in the same course. Overall, the maximum diameters of these lesions treated with SBRT were < 1 cm (15%), 1.1 to 2 cm (50%), 2.1 to 3 cm (25%), and > 3 cm (11%). Seventeen tumors were immobilized with S-frame and 76 with BodyFIX.

Patient and tumor characteristics were generally balanced between immobilization arms (Table 1), where no significant differences were present except for BSA ($p = 0.03$; Table 1). The S-frame group had significantly more right-sided tumors ($p = 0.02$). Forty-one lesions (44%) were biopsy-confirmed NSCLC (adenocarcinoma most commonly), 15 (16%) were metastatic from another site, and 37 (40%) were not biopsy proven.

Table 1. Characteristics of 88 patients and 93 tumors

| *Characteristic*[a] | *BodyFIX* | *S-Frame* | P-*Value* |
|---|---|---|---|
| Age at diagnosis, yr | | | |
| Mean (SD) | 70.7 (9.9) | 68.9 (12.0) | 0.53 |
| Male sex | 37 (50.7) | 4 (26.7) | 0.09 |
| Body mass index, kg/m$^2$ | | | |
| Mean (SD) | 28.4 (7.1) | 26.2 (5.7) | 0.47 |
| Body surface area, m$^2$ | | | |
| Mean (SD) | 1.9 (0.3) | 1.8 (0.2) | 0.03 |
| COPD status and severity | | | |
| No COPD | 31 (42.5) | 7 (46.6) | |
| Mild | 9 (12.3) | 2 (13.3) | |
| Moderate | 11 (15.0) | 1 (6.7) | 0.23 |
| Severe | 11 (15.0) | 4 (26.7) | |
| Oxygen-dependent | 11 (15.0) | 1 (6.7) | |
| Smoking status | | | |
| Current | 6 (8.2) | 2 (13.3) | |
| Never | 21 (28.8) | 1 (6.7) | 0.19 |
| Past | 46 (63.0) | 12 (80.0) | |
| Laterality | | | |
| Left | 37 (48.7) | 3 (17.6) | 0.02 |
| Right | 39 (51.3) | 14 (82.4) | |
| Centrality | | | |
| Central | 18 (23.7) | 3 (17.6) | 0.59 |
| Peripheral | 58 (76.3) | 14 (82.4) | |
| Lesion size, cm | | | |
| Median (range) | 1.8 (0.5-6.6) | 1.5 (0.7-2.3) | 0.07 |
| Tumor histologic findings | | | |
| NSCLC | 33 (43.4) | 8 (47.1) | |
| Metastatic | 13 (17.1) | 2 (11.8) | 0.86 |
| Not biopsy proven | 30 (39.5) | 7 (41.2) | |
| Clinical T stage | | | |
| 1A | 28 (36.8) | 8 (47.1) | |
| 1B | 14 (18.4) | 1 (5.9) | 0.53 |
| 2A | 7 (9.2) | 0 (0.0) | |
| 3 | 1 (1.3) | 0 (0.0) | |

[a] Values are presented as number and percentage of patients, unless specified otherwise.
COPD = chronic obstructive pulmonary disease; NSCLC = non–small cell lung cancer.

## B. Treatment characteristics and outcome

SBRT doses to the planning target volume were 34 Gy/1 fraction (n = 1 [1%]), 48 Gy/4 fractions (n = 26 [28%]),[9] 50 Gy/5 fractions (n = 22 [24%]), and 54 Gy/3 fractions (n = 43 [46%]). At the end of follow-up, 87 (95.6%) of 91 lesions (two cases were not evaluable) were locally controlled. Although all four locally recurrent cases were in patients immobilized with BodyFIX, the difference between the two arms was not significant (94.6% vs. 100%; $p = 0.33$). For all patients, two- and three-year LC rates were 94% and 87%, respectively; median was not reached yet. No difference in LC was found for patients immobilized with S-frame versus BodyFIX (log-rank $p = 0.35$). PFS was calculated for the 56 patients (63.6%) who had primary or recurrent disease, for which SBRT intent was curative. At end of follow-up, patients with 16 (24%) of the 67 evaluable lesions had progression locally or distally or had died, whichever occurred earlier; development of distant metastatic lung cancer was a common pattern of treatment failure. Seventy-two patients (81.8%) were alive at the end of follow-up. No difference was seen in LC by age, PFT parameters, body habitus, COPD, or smoking status, though tumors > 2 cm had poorer LC in final multivariate analysis (log-rank $p < 0.006$). LC, PFS, and overall survival were not significantly different (all log-rank $p > 0.35$) for S-frame versus BodyFIX, an expected result.

## C. Immobilization and patient factors

In total, 347 fractions (65 S-frame and 282 BodyFIX) were delivered. First couch shifts were significantly lower with S-frame than BodyFIX for F1 through F3. S-frame strategy improved patient immobilization in magnitude of shift errors (Table 2) and number of shifts required (mainly driven by F1), translating into fewer CBCT scans per patient. First couch shift by CBCT exceeded the 4 mm tolerance in 33 fractions (51%) in S-frame versus 217 (77%) in BodyFIX ($p < 0.001$). Correction through first couch shifts was usually successful; second couch shifts were similarly small, regardless of immobilizing device; third shifts were rarely needed. First shift differences of first (F1, S1), second (F2, S1), and third (F3, S1) fractions were significant (all $p \leq 0.03$), favoring smaller shifts in S-frame cases. Figure 2 shows the box plot for first shift of first fraction (F1, S1) error with BodyFIX versus S-frame. S-frame significantly decreased shift errors by CBCT in univariate and multivariate analyses ($p < 0.001$).

Patient-related factors had substantial impact on the robustness of SBRT setup. Table 3 shows summary and analysis of the PFT variables examined. PFT values were balanced between the immobilization arms. No significant correlation was found in BMI comparison (≤ 25 vs. > 25) across all fractional and shift error variables; BSA was not associated with the first shift of first ($p = 0.37$) or second ($p = 0.08$) fractions. In univariate analyses, normalized $FEV_1$ was significant for both first shift of first fraction ($p < 0.04$) and averaged shift error in first fractions ($p < 0.04$).

Table 2. Summary of interfractional errors measured with 3D couch shift parameters

| *3D Calculation of CBCT Shifts* | *Immobilization Choice Mean (SD) (cm)* | | |
|---|---|---|---|
| | *BodyFIX* | *S-Frame* | P-*Value* |
| Fraction 1, shift 1 | 1.2 (0.7) | 0.4 (0.3) | <0.001 |
| Fraction 1, shift 2 | 0.2 (0.4) | 0.3 (0.2) | 0.02 |
| Fraction 1, shift 3 | 0.1 (0.1) | 0.1 (0.0) | >0.99 |
| Fraction 2, shift 1 | 1.0 (0.7) | 0.6 (0.3) | 0.01 |
| Fraction 2, shift 2 | 0.2 (0.2) | 0.3 (0.3) | 0.13 |
| Fraction 3, shift 1 | 1.0 (0.8) | 0.6 (0.4) | 0.03 |
| Fraction 3, shift 2 | 0.1 (0.1) | 0.3 (0.2) | 0.06 |
| Fractions 2-5, shift 3 | 0.2 (0.1) | 0.1 (0.1) | 0.95 |

CBCT = cone-beam computed tomography; Fractions 2-5 = fractions second through fifth, averaged; 3D = three-dimensional.

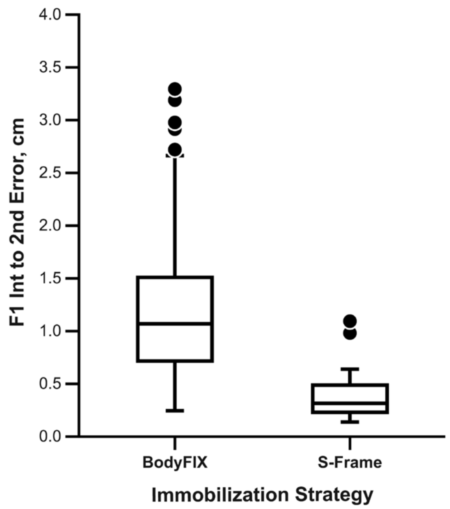

Fig. 2. Box plots for first fraction (F1) and first shift errors with BodyFIX vs. S-frame immobilization strategies (both $p < 0.001$). Error bars = SDs; solid dots = outliers greater than 1 SD in magnitude; Int = initial.

TABLE 3. Pulmonary function test summary of the 88 study patients

| *Test* | *Immobilization Choice Mean (SD) (cm)* *BodyFIX* | *S-Frame* | P-*Value* |
|---|---|---|---|
| Total lung capacity, L | 6.2 (1.9) | 5.7 (1.7) | 0.63 |
| Vital capacity, L | 2.8 (1.1) | 2.3 (0.8) | 0.24 |
| Residual volume, L | 3.6 (1.6) | 3.4 (1.3) | 0.88 |
| $FEV_1$, L | 1.6 (1.0) | 1.3 (0.7) | 0.28 |
| $FEV_1$% | 0.6 (0.3) | 0.5 (0.3) | 0.42 |
| Normalized ratio of $FEV_1$ to FVC | 0.7 (0.2) | 0.7 (0.2) | 0.99 |
| DLCO, mL CO per min, mm Hg | 12.2 (6.4) | 10.9 (4.9) | 0.63 |

CO = carbon monoxide; DLCO = diffusing capacity for carbon monoxide; $FEV_1$ = forced expiratory volume in first second of expiration; $FEV_1$% = forced expiratory volume in first second of expiration, percent predicted; FVC = forced vital capacity.

Absolute $FEV_1$ was inversely related to the initial shift error occurring in the first fraction. An $FEV_1 \leq 1.5$ L caused a mean (SD) shift error of 1.2 (0.7) cm compared with an $FEV_1 > 1.5$ L causing 0.9 (0.6) cm ($p < 0.04$), regardless of immobilization choice.

## D. Multivariate analyses

Patient immobilization choice, body habitus, and PFT values were used in a multistep, multivariate analysis for modeling the interfractional errors estimated. Table 4 shows stepwise linear models for interfractional errors by immobilization, with or without BMI or BSA. Interfractional errors were fitted to immobilization (Immobn) choice (Immobn, binary; BodyFIX, 0; S-frame, 1), BMI (continuous), or BSA (continuous):

$$F1_{\text{averaged}} = \text{Immobn}_{\text{param}} \times [\text{BodyFIX} = 0; \text{S-frame} = 1] + \text{BSA}_{\text{param}} \times [\text{BSA}] + \text{Intercept} \qquad (1)$$

This equation shows an example of how errors were fitted,(1) with "param" indicating parameter.

First shift of first fraction, averaged shift of first fraction, averaged shift of second fraction, and averaged shift of all fractions were well correlated ($p < 0.005$) to immobilization choice and BSA (or BMI). Importantly, the number of CBCT scans required in the first fraction correlated well to immobilization choice alone, with a mean (SD) CBCT of 0.49 (0.12) less per patient for S-frame; the average number of CBCT scans required for BodyFIX was 1.96 before the tumor was successfully aligned for SBRT. In the subsequent modeling step, PFT values were also subsequently significant in predicting shift errors across first and second fractions. A separate multivariate model showed that the first shift of first fraction significantly correlated with immobilization choice and the normalized ratio of $FEV_1$ to FVC ($F = 8.66$; $p < 0.001$). Averaged shift in first fraction similarly correlated with these two variables ($F = 7.68$; $p < 0.001$). Figure 3 shows a scatter plot matrix of selected variables (i.e., body habitus and pulmonary function parameters).

TABLE 4. Multivariate modeling of interfractional errors with patient immobilization mode, BMI, or BSA, or a combination

| *Multivariate Analysis*[a] | *S-Frame vs. BodyFIX*[b,c] | *BMI*[c] | *BSA*[c] | *Intercept* | *Model Pr>F* |
|---|---|---|---|---|---|
| First fraction, first shift (F1, S1) | –0.756 (0.177) | N/A | 0.370 (0.241) | 0.5160 | <0.0001 |
| First fraction, averaged shift (F1_averaged) | –0.309 (0.095) | N/A | 0.297 (0.130) | 0.1418 | <0.0001 |
| Second fraction, averaged shift (F2_averaged) | –0.206 (0.098) | 0.0136 (0.006) | N/A | 0.2579 | 0.0049 |
| All fractions, averaged shift (F_all_averaged) | –0.176 (0.062) | N/A | 0.273 (0.085) | 0.1091 | <0.0001 |
| First fraction, No. of CBCT (F1_number) | –0.490 (0.123) | N/A | N/A | 1.9605 | <0.0001 |

[a] Linear multivariate model: Error, cm = [BodyFIX = 0; S-frame = 1] × Immobilization_param + BSA × BSA_param (or BMI × BMI_param) + Intercept. The Intercept value (error in cm or number of CBCT) was modeled when other relevant independent parameters were held to zero.
[b] BodyFIX = 0; S-frame = 1.
[c] Values are provided as coefficient for the selected parameter (SE).
BMI = body mass index; BSA = body surface area; CBCT = cone-beam computed tomography; N/A = not applicable; Pr>F = probability greater than F value.

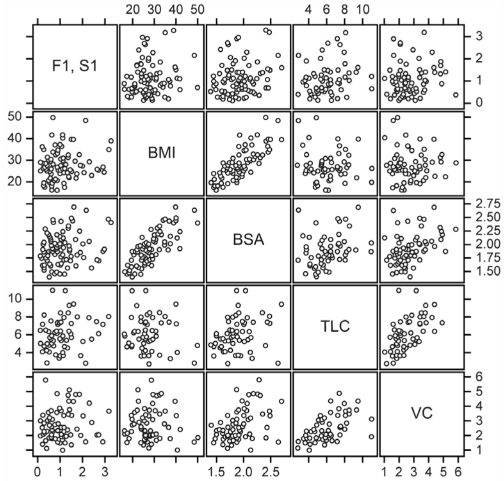

FIG. 3. Scatter plot matrix for variable interaction; each scatter plot subbox represents graphing of the two variables compared. For example, the box in the first row and second column represents F1, S1 (y-axis) vs. BMI (x-axis). BMI = body mass index, kg/m$^2$; BSA = body surface area per m$^2$; F1, S1 = first fraction, first shift, cm; TLC = total lung capacity, L; VC = vital capacity, L.

## IV. DISCUSSION

Our study showed that both choice of immobilization and physiological factors, including baseline pulmonary function and body habitus, had a strong role in determining setup accuracy for SBRT. Our results are in agreement with two other studies that also used BodyFIX.[(7,10)] A 2009 study from Baba et al.[(10)] reported that for 55 lesions, use of BodyFIX significantly reduced tumor movements during free-breathing condition. Han et al.[(7)] compared 24 patients undergoing SBRT for medically inoperable stage I NSCLC or pulmonary metastases. Patients were assigned prospectively and randomly to BodyFIX or abdominal compression plate.

Of 25 lesions, 16 (64%) were upper lobe tumors. The investigators noted a range of 4.6 to 5.3 mm of tumor motion for BodyFIX and 5.3 to 6.1 mm during free breathing. Their use of four-dimensional CT simulation and CBCT scans was identical to our practice and should be reinforced. The magnitudes of recorded tumor motion also were comparable to the results of our study.(11)

Additional studies have been performed to quantify positional change in lung lesions during SBRT setup with CBCT. Yeung et al.(12) assessed 13 lung cancer patients for whom CBCT was used in daily image guidance as the patients underwent conventional external beam radiotherapy. Seven patients had T1 to T3 upper lung tumors. BMIs of these seven patients ranged from 17.2 to 25.6 $kg/m^2$. Taken together, they noted that systemic and random errors when CBCT was not used were up to 3.2 to 5.6 and 2.0 to 3.5 mm, respectively, which was most apparent in the craniocaudal direction (mean, 3.9 mm). With CBCT, error rate in all directions could be reduced to 0.3 to 1.1 mm. Negoro et al.(13) evaluated setup accuracy of solitary lung lesions undergoing hypofractionated radiotherapy. Using port films and CT images, they noted that tumor movement during respiration ranged from 0 to 2 cm; however, a 90% success rate was noted for limiting daily setup errors to within 5 mm in all directions. These studies highlighted the importance of respiratory motion management, especially for thoracic SBRT in which highly precise radiotherapy delivery is required.

Matsugi et al.(14) quantified interfractional variations in gross tumor volume of eight patients who underwent SBRT for lung cancer. Four patients had upper lung tumors. Although random variations in gross tumor volume motion were small in upper lobe tumors (within 1.0 mm), interfractional variations were larger, especially in anteroposterior (1.4–1.8 mm) and craniocaudal (1.3–1.9 mm) directions. Similarly, Purdie et al.(15) noted that target localization was highly accurate, but cautioned that simply relying on bony anatomy correlation, such as orthogonal on-board imaging, may result in mismatch and false registration of the tumor. For larger tumors (mean [SD]: 5.5 [3.1] cm) with conventional external beam irradiation (not SBRT), Stevens et al.(16) showed that respiratory movement of lung lesions was not associated with tumor size, location, or PFT, which differed from our SBRT results. Four of their five upper lung tumors showed significant superior–inferior displacement in their study. The difference in results between the two studies may be due to the fact that Stevens and colleagues applied orthogonal radiographs instead of modern CBCTs. In addition, their number of evaluated patients was small (22 patients vs. 88 patients in the present study). Our data complemented the current literature in thoracic oncology as a result.

In early-stage NSCLC, if $FEV_1$ or diffusing capacity of lung for carbon monoxide is ≤ 40% predicted, patients are unlikely to be candidates for surgical resection. A postoperative $FEV_1$ between 30% and 40% has been shown to increase postoperative complications.(17) In our patient population, about 40% to 60% would be ineligible for surgery. Other existing medical comorbidities (e.g., coronary artery disease, diabetes mellitus) may have a role in medical decision making. Interestingly, our data and multivariate models suggested that poorer $FEV_1$ (and poorer normalized ratio of $FEV_1$ to FVC) was significantly correlated to increased setup error generated during the initial shift incurred in the first fraction of the first treatment day. Because SBRT required lying flat for a long time during setup and treatment, further respiratory couching or oxygen supplementation may be beneficial. Decline in pulmonary function was small after SBRT to the lung, even for morbid and COPD patients.(18,19)

An important consideration for the adapted use of S-frame immobilization strategy is that potentially, patient comfort can be improved. The patient's overall comfort during the SBRT procedure, and also the ease of setup by radiotherapists, may be a factor in minimizing respiratory motion during treatment. It is conceivable that patients immobilized with S-frame were more comfortable during setup and treatment than patients who were immobilized with BodyFIX. Future prospective, patient-oriented surveys about the treatment experience may give us more information on this topic.

Limitations of our study include retrospective study design and heterogeneous patient population (i.e., patients with primary and recurrent lung cancer diagnoses and those with metastatic disease to the lung). However, the study focus was not clinical outcome, but rather the robustness of daily clinical setup for patients undergoing SBRT. The result is not generalizable to parts of the lungs outside the upper region. The number of patients immobilized with S-frame was smaller than with BodyFIX; as a result, it is possible that smaller, more subtle differences in other shift changes or physiologic parameters may go undetected. A larger study similar to this one will be desirable in validating our findings in the future.

Of note, the present study was not designed for investigating errors caused by traditional anatomical matching, such as orthogonal on-board imaging. On the basis of clinical experience, pretreatment on-board images were acquired on first treatment day and matched before CBCT. For subsequent fractions, only CBCT was used. This imaging procedure was used for both immobilizations, BodyFIX and S-frame. For shift analysis, we excluded on-board image shifts by comparing the initial couch position with the one after CBCT alignment. Hence, they were not included in all analyses performed in this study.

Of importance, we showed that the number of CBCT scans used in SBRT can be decreased. This reduction can be accomplished through choosing the proper immobilization device for the region of interest for treatment, while taking into account pulmonary reserve and body habitus during irradiation and dosimetric planning.

## V. CONCLUSIONS

For upper lung tumors treated with SBRT, initial setup was more consistent with S-frame than BodyFIX, which resulted in fewer CBCT scans. Patients with obese habitus and poorer pulmonary function had more SBRT setup uncertainty with CBCT. Thus, medical physicists and clinical radiation oncologists must be prepared to account for greater uncertainty in radiotherapy setup and target planning for these patients. We advocate the use of S-frame for immobilizing patients receiving SBRT to the upper lung.

## Supporting information

Supplementary Material

## References

[1] Timmerman R , Paulus R , Galvin J et al. Stereotactic body radiation therapy for inoperable early stage lung cancer. JAMA. 2010;303(11):1070–76. 20233825 10.1001/jama.2010.261 PMC2907644

[2] Ricardi U , Filippi AR , Guarneri A et al. Stereotactic body radiation therapy for lung metastases. Lung Cancer. 2012;75(1):77–81. 21726918 10.1016/j.lungcan.2011.04.021

[3] Fritz P , Kraus HJ , Muhlnickel W et al. Stereotactic, single‐dose irradiation of stage I non‐small cell lung cancer and lung metastases. Radiat Oncol. 2006;1:30. 16919172 10.1186/1748-717X-1-30 PMC1579222

[4] Wulf J , Haedinger U , Oppitz U , Thiele W , Mueller G , Flentje M . Stereotactic radiotherapy for primary lung cancer and pulmonary metastases: a noninvasive treatment approach in medically inoperable patients. Int J Radiat Oncol Biol Phys. 2004;60(1):186–96. 15337555 10.1016/j.ijrobp.2004.02.060

[5] Stephans K . Stereotactic body radiotherapy for stage I non‐small cell lung cancer. Cleve Clin J Med. 2012;79 Electronic Suppl 1:eS26–e531. 22614962 10.3949/ccjm.79.s2.06

[6] Bennouna J , Breton JL , Tourani JM et al. Vinflunine: an active chemotherapy for treatment of advanced non‐small‐cell lung cancer previously treated with a platinum‐based regimen: results of a phase II study. Br J Cancer. 2006;94(10):1383–88. 16641911 10.1038/sj.bjc.6603106 PMC2361262

[7] Han K , Cheung P , Basran PS , Poon I , Yeung L , Lochray F . A comparison of two immobilization systems for stereotactic body radiation therapy of lung tumors. Radiother Oncol. 2010;95(1):103–08. 20189669 10.1016/j.radonc.2010.01.025

[8] Stauder MC , Macdonald OK , Olivier KR et al. Early pulmonary toxicity following lung stereotactic body radiation therapy delivered in consecutive daily fractions. Radiother Oncol. 2011;99(2):166–71. 21571384 10.1016/j.radonc.2011.04.002

[9] Nagata Y , Takayama K , Matsuo Y et al. Clinical outcomes of a phase I/II study of 48 Gy of stereotactic body radiotherapy in 4 fractions for primary lung cancer using a stereotactic body frame. Int J Radiat Oncol Biol Phys. 2005;63(5):1427–31. 16169670 10.1016/j.ijrobp.2005.05.034

[10] Baba F , Shibamoto Y , Tomita N et al. Stereotactic body radiotherapy for stage I lung cancer and small lung metastasis: evaluation of an immobilization system for suppression of respiratory tumor movement and preliminary results. Radiat Oncol. 2009;4:15. 19476628 10.1186/1748-717X-4-15 PMC2694202

[11] Sonke JJ , Rossi M , Wolthaus J , van Herk M , Damen E , Belderbos J . Frameless stereotactic body radiotherapy for lung cancer using four‐dimensional cone beam CT guidance. Int J Radiat Oncol Biol Phys. 2009;74(2):567–74. 19046825 10.1016/j.ijrobp.2008.08.004

[12] Yeung AR , Li JG , Shi W et al. Tumor localization using cone‐beam CT reduces setup margins in conventionally fractionated radiotherapy for lung tumors. Int J Radiat Oncol Biol Phys. 2009;74(4):1100–07. 19395197 10.1016/j.ijrobp.2008.09.048

[13] Negoro Y , Nagata Y , Aoki T et al. The effectiveness of an immobilization device in conformal radiotherapy for lung tumor: reduction of respiratory tumor movement and evaluation of the daily setup accuracy. Int J Radiat Oncol Biol Phys. 2001;50(4):889–98. 11429216 10.1016/s0360-3016(01)01516-4

[14] Matsugi K , Narita Y , Sawada A et al. Measurement of interfraction variations in position and size of target volumes in stereotactic body radiotherapy for lung cancer. Int J Radiat Oncol Biol Phys. 2009;75(2):543–48. 19735879 10.1016/j.ijrobp.2008.12.091

[15] Purdie TG , Bissonnette JP , Franks K et al. Cone‐beam computed tomography for on‐line image guidance of lung stereotactic radiotherapy: localization, verification, and intrafraction tumor position. Int J Radiat Oncol Biol Phys. 2007;68(1):243–52. 17331671 10.1016/j.ijrobp.2006.12.022

[16] Stevens CW , Munden RF , Forster KM et al. Respiratory‐driven lung tumor motion is independent of tumor size, tumor location, and pulmonary function. Int J Radiat Oncol Biol Phys. 2001;51(1):62–68. 11516852 10.1016/s0360-3016(01)01621-2

[17] Mazzone P . Preoperative evaluation of the lung resection candidate. Cleve Clin J Med. 2012;79 Electronic Suppl 1:eS17–e522. 22614960 10.3949/ccjm.79.s2.04

[18] Stephans KL , Djemil T , Reddy CA et al. Comprehensive analysis of Pulmonary Function Test (PFT) changes after stereotactic body radiotherapy (SBRT) for stage I lung cancer in medically inoperable patients. J Thorac Oncol. 2009;4(7):838–44. 19487961 10.1097/JTO.0b013e3181a99ff6

[19] Takeda A , Enomoto T , Sanuki N et al. Reassessment of declines in pulmonary function ≥ 1 year after stereotactic body radiotherapy. Chest. 2013;143(1):130–37. 22722234 10.1378/chest.12-0207

