# Supplementary material for: Influence of patient's physiologic factors and immobilization choice with stereotactic body radiotherapy for upper lung tumors
Source: J Appl Clin Med Phys. 2014 Sep 8;15(5):235–45. doi: 10.1120/jacmp.v15i5.4931 (PMC5711083; doi:10.1120/jacmp.v15i5.4931)
Supplement: Supplementary file 1 — Supplementary Material [file ACM2-15-235-s001.doc]

[Category: Original Article]

Patient Immobilization With Stereotactic Body Radiotherapy for Upper Lung Tumors

Terence T. Sio, MD, MS

Andrew R. Jensen, MS

Robert C. Miller, MD, MS

Luis E. Fong de los Santos, PhD

Christopher L. Hallemeier, MD

Nathan R. Foster

Sean S. Park, MD, PhD

Heather J. Bauer, RN, CNP

Yolanda I. Garces, MD

Kenneth R. Olivier, MD

**Author Affiliations:** Department of Radiation Oncology (Drs Sio, Miller, Fong de los Santos, Hallemeier, Park, Garces, and Olivier, Mr Jensen, and Ms Bauer), and Division of Biomedical Statistics and Informatics (Mr Foster), Mayo Clinic, Rochester, Minnesota.

**Reprints:** Kenneth R. Olivier, MD, Department of Radiation Oncology, Mayo Clinic, 200 First St SW, Rochester, MN 55905 (olivier.kenneth@mayo.edu).

Presented at the 54th annual meeting of the American Society for Radiation Oncology, Boston, Massachusetts, October 28-31, 2012.

Conflict of interest: None.

Text word count: 2,728

Abstract word count: 297

No. of tables: 4

No. of figures: 2

Running title: Setup in Upper Lung Tumor SBRT

**Publisher:** To expedite proof approval, send proof via e-mail to scipubs@mayo.edu.

©2014 Mayo Foundation for Medical Education and Research
